# Small intestine intussusception causing intestinal obstruction secondary to left pinna melanoma metastases: a case report

**DOI:** 10.3389/fsurg.2026.1885354

**Published:** 2026-09-24

**Authors:** Jorge Luis Turizo Farias, Rogers Leonardo Baquero, Samir Leonardo Moreno-Martinez, Sergio Alberto Lesmes, Sofia Molano Rodriguez, Juana Gabriela Rueda, Isabella Van-Londoño

**Affiliations:** 1 Department of Surgery, Hospital Universitario Mayor-Méderi, Bogotá, Colombia; 2 School of Medicine and Health Sciences, Universidad del Rosario, Bogotá, Colombia

**Keywords:** gastrointestinal tumor, intestinal obstruction, intestinal resection and anastomosis, melanoma, metastatic melanoma

## Abstract

We present the case of a 68-year-old patient with a previous history of stage IV malignant ulcerated nodular melanoma of the left pinna with pulmonary metastasis, who was admitted to the emergency department with an initial clinical suspicion of intestinal obstruction, which was confirmed by computed tomography. He underwent an exploratory laparotomy that revealed multiple small lesions in the small intestine suggestive of malignancy, causing a distal jejunal intussusception responsible for the obstruction. The patient underwent resection of the affected bowel segment and subsequent intestinal anastomosis, with histopathological samples confirming metastatic melanoma. The patient progressed with an adequate postoperative course and is now on a systemic oncology management plan. This case study reports an infrequent presentation of left pinna melanoma intestinal metastases presenting with small bowel obstruction and successful surgical management given to the patient to further broaden knowledge on this subject.

## Introduction

Melanoma is a type of epithelial cancer that originates from melanocytes. It is found in multiple types of tissues but is predominantly present in the integumentary system. Although it is the rarest form of skin cancer, it is the deadliest and the one that carries the highest risk of metastatic disease (1). Gastrointestinal tissue metastases are one of the most common types of metastases found in this type of cancer, being positive in up to 60% of autopsies performed on patients suffering from this disease. However, clinical manifestations account for only of 1%–5% of cases, having affinity to the small bowel (51%–71%), especially the ileum and jejunum, followed by the stomach (27%), large intestine (22%), and esophagus (5%) (2–4). Symptoms are similar to those of other gastrointestinal tumors, including abdominal pain, intestinal obstruction, bowel perforation, hematemesis, and melena.

We present the case of a patient with a previous history of left pinna melanoma managed with neoadjuvant chemoradiotherapy followed by surgical resection, who presented months later with abdominal pain and intestinal obstruction caused by metastatic lesions confirmed by histopathology, requiring resection of the affected bowel segment.

## Case presentation

We present the case of a 68-year-old patient with a history of malignant, nodular, ulcerated melanoma of the left auricular pavilion (stage IIIc, BRAF-mutated with a 60% Ki67 index) diagnosed 5 years ago during an ultrasound follow-up. Due to the finding of a suspicious left parathyroid adenomegaly, the patient underwent a partial auriculectomy and left parathyroidectomy with lymphadenectomy. The operative note described a nodular 1.5-cm×1-cm lesion involving the posterior wall of the helix, with satellite lesions located 0.5 cm both superior and inferiorly. Afterward, the patient received maintenance immunotherapy with pembrolizumab. Three years later, during a follow-up appointment, the patient underwent a thoracoscopic lung wedge resection for a nodular lesion in the lingula. Histopathological examination confirmed metastatic melanoma (MM) with a 60% Ki67 index and positive immunohistochemistry for S100, SOX10, HMB45, and Melan-A, updating the patient's disease stage to IV.

During oncological follow-up, the patient reported intermittent abdominal pain. An abdominal magnetic resonance imaging scan revealed a nodular small lesion, which was asymptomatic but managed with strict observation given the recent pulmonary metastasis diagnosis. Two months after the lung wedge resection, the patient was admitted to the emergency department due to emesis, pain, and abdominal bloating, and was subsequently taken to an abdominal computed tomography scan documenting ascites and small bowel distended loops with a transition zone in the distal jejunum, suggesting intestinal obstruction (Figure 1).

**Figure 1:**
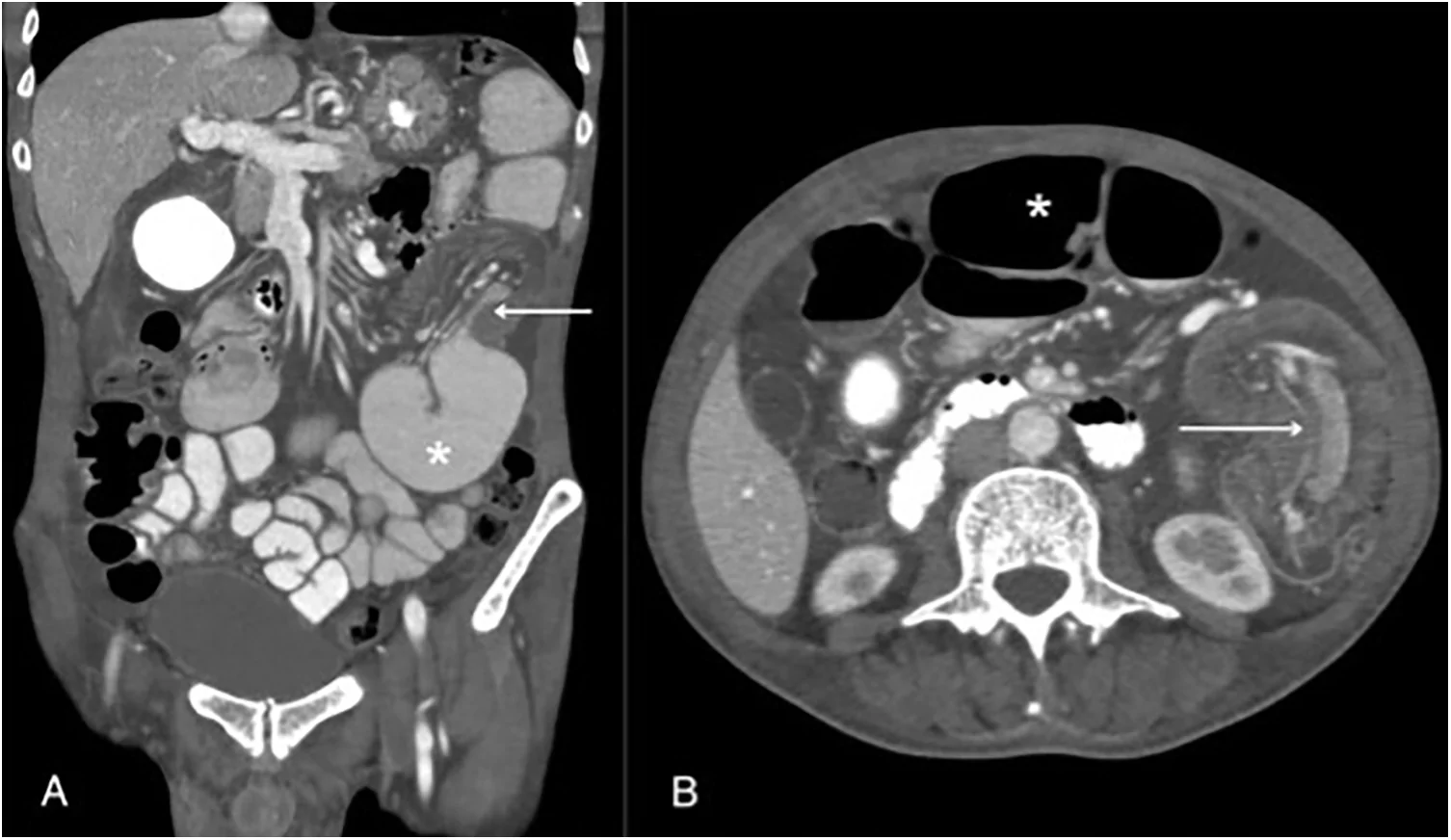
Computed tomography findings suggesting intestinal intussusception, considering proximal bowel loop distention with a sudden transition zone (white arrow and asterisk), in coronal **(A)** and axial **(B)** planes.

Due to these findings, the patient underwent an exploratory laparotomy, which revealed abundant serous ascitic peritoneal fluid and a 30-cm segment of intussuscepted distal jejunum caused by an indurated, nodular, ulcerated lesion with transmural inflammation originating from the small bowel wall (Figure 2), resulting in retrograde distention of the intestinal loops. When reducing this intussusception, three solid and ulcerated lesions were seen at 40, 50, and 80 cm distal to the Treitz ligament. Considering the above, a segmental 100-cm small-bowel resection with a manual latero-lateral anastomosis was performed to restore new continuity of the gastrointestinal tract (GIT) 30 cm distal to the fixed loop, without complications.

**Figure 2:**
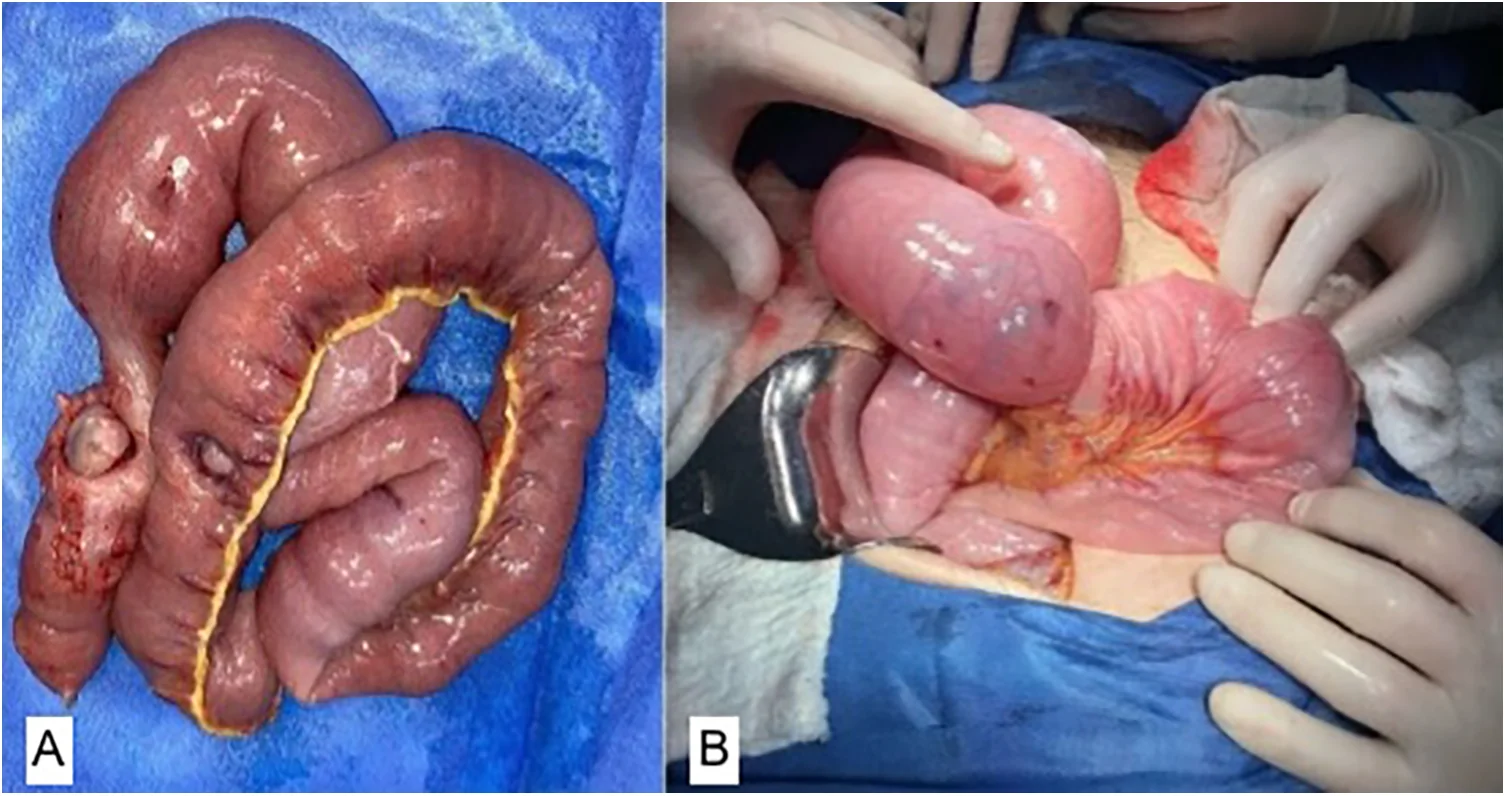
Intraoperative findings. **(A)** Resected intestinal segment *en bloc*. **(B)** Intestinal bowel segment that was intussuscepted.

The patient had an adequate postoperative course without any acute complications, demonstrated appropriate intestinal transit, and was discharged from the hospital. The histopathological report documented metastatic melanoma involvement by transmural distribution of multiple amelanocytic lesions with intestinal wall ulceration. The patient was then referred for an oncology consultation and was indicated for treatment with dabrafenib and trametinib. The patient reports satisfaction with the treatment given and is currently awaiting targeted therapy. A flowchart depicting the disease chronology of the patient is shown in Figure 3. Specific dates were avoided to ensure patient confidentiality.

**Figure 3:**
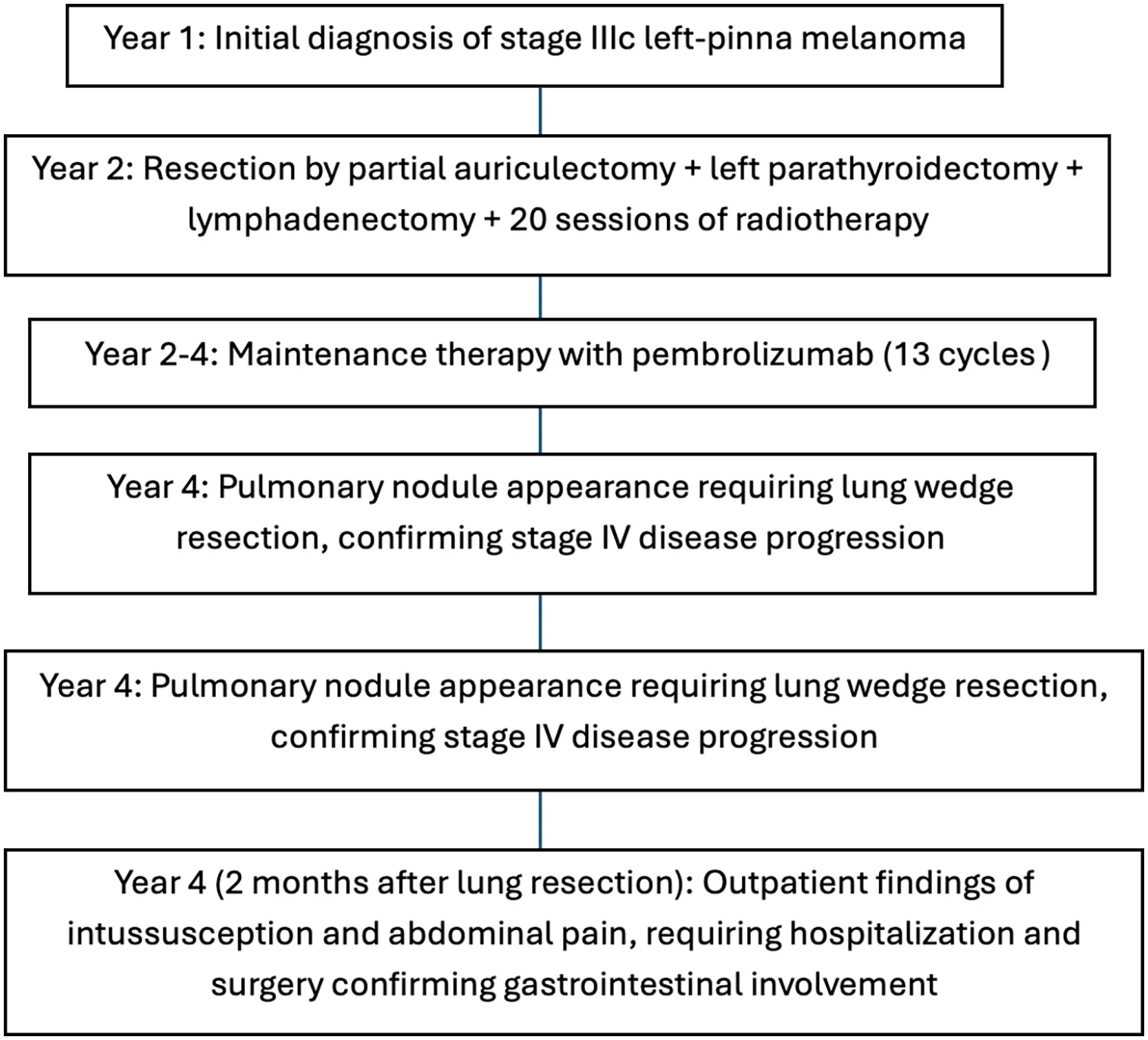
Flowchart showing chronology and disease evolution. Patient disease chronology by years.

## Discussion

Melanoma involving the GIT are among the most common types of metastases in this type of tumor, with an approximate incidence of 43.5% (5, 6). These lesions usually present as multiple ulcerated, polypoid masses that can be pigmented or amelanocytic. They can manifest concurrently with the diagnosis of the primary tumor or emerge even decades later as the initial sign of recurrent disease. Symptoms are non-specific and can mimic a variety of GIT pathologies, including abdominal pain, fatigue, dysphagia, constipation, small bowel perforation, gastrointestinal bleeding, or anemia. When there is small bowel involvement, a percentage of patients can present with intussusception or a retroperitoneal mass and are, in most cases, susceptible to surgical treatment, as was the case in our patient (7, 8).

The diagnosis of MM must be guided by clinical presentation, with the aid of imaging studies such as computed tomography (with a sensitivity of 60%–70%) or endoscopic and barium contrast studies. Positron-emission tomography has recently been used to identify possible sites of metastases (93% sensitivity); however, a strong clinical suspicion must guide the indication for this study (9, 10).

The molecular characteristics of this type of tumor (cutaneous melanoma) include oncogenic mutations such as BRAF (serine/threonine kinase protein) and NRAS (neuroblastoma viral RAS oncogene). Approximately half of patients with MM have a BRAF mutation, of which 90% are BRAFV600 mutations and are susceptible to treatment with BRAF and MEK inhibitors (BRAFi/MEKi) (11, 12).

GIT MM treatment includes surgical resection, radiotherapy, and targeted systemic therapy, including chemotherapy, immunotherapy, and biochemotherapy. However, multiple studies suggest that complete surgical resection of GIT metastatic lesions offers better symptom control and is associated with better prognosis and disease-free survival (13). Ollila et al. evaluated 124 patients who were candidates for surgical resection of MM metastasis of the GIT (excluding esophageal lesions), documenting that 97% of patients undergoing surgical treatment (67 of 69 patients) experienced immediate postoperative symptom relief, with a mean survival time of 48.9 months, which is drastically higher than the 5.4–5.7 months in patients receiving palliative non-surgical treatment (13, 14). Similar findings were reported in a 2002 retrospective study by Panagitou et al., which evaluated 18 patients with GIT metastases from a cohort of 385, with results demonstrating a median survival of 47.5 months in patients treated surgically, compared with only 5.8 months in the medical group (15). This is especially important given that chemotherapy has not yielded the best results and that current treatment with dacarbazine for such patients has a very low adequate response rate (only around 5%–28% of patients) (16). Conversely, immunotherapy has revolutionized successful treatment for this type of tumor, as it is possible to achieve a higher response rate with immune checkpoint inhibitors (ICIs), including ipilimumab, tremelimumab, nivolumab, pembrolizumab, atezolizumab, avelumab, and durvalumab, and BRAFi/MEKi such as dabrafenib and trametinib. Current guidelines on melanoma recommend systemic therapy with pembrolizumab for patients with initially resected primary tumors, as in the initial case of our patient. However, when disease progresses to resectable stage IV, combined adjuvant therapy with nivolumab–ipilimumab or dabrafenib and trametinib, despite limited evidence, is the most recommended therapy and is one of the treatment options our patient was awaiting (17). Overall evidence suggests that when targeted therapies are used in combination, they drastically improve survival in BRAF-positive MM patients, with current clinical treatments reporting adjuvant treatment with ICIs and BRAFi/MEKi as the current standard of care for patients with advanced-stage disease undergoing surgical resection (18, 19).

Considering all the above, patients with a history of melanoma consulting for non-specific gastrointestinal symptoms must have metastatic disease as one of the main differential diagnoses, considering its predisposition to this type of tissue. Diagnosis depends on clinical presentation, with multiple diagnostic tools at the physician's disposal, such as endoscopic and contrast imaging studies. In recent years, treatment and prognosis have drastically improved thanks to various oncologically directed precision treatments such as ICIs and BRAFi/MEKi. The combination of surgical resection and current effective systemic treatments, such as the one provided to our patient, should be considered to further improve short- and long-term survival in this group of patients.

Limitations of this case report include the inability to perform a prior positron emission tomography scan due to institutional unavailability and the lack of any follow-up data on the patient after starting immunotherapy. The authors have completed the CARE Checklist for this case report, and it is attached as Supplementary Material.

## Data Availability

The original contributions presented in the study are included in the article/Supplementary Material; further inquiries can be directed to the corresponding author.
